# CHARACTERISTICS AND CARE EXPERIENCES OF OLDER DUAL-ENROLLEES LIVING IN AREAS WITH MEDICAID MANAGED LTSS

**DOI:** 10.1093/geroni/igad104.2038

**Published:** 2023-12-21

**Authors:** Andrew Jopson, Chanee Fabius, Jennifer Wolff

**Affiliations:** Johns Hopkins University, Baltimore, Maryland, United States; Johns Hopkins University, Baltimore, Maryland, United States; Johns Hopkins University, Baltimore, Maryland, United States

## Abstract

Medicaid-funded long-term services and supports (LTSS) are foundational in helping older dual-enrollees with disabilities remain in the community. Over the past decade, nearly half of U.S. states have transformed LTSS delivery through managed LTSS (MLTSS) with the goal of improving care coordination and avoiding unnecessary health care utilization. Despite this rapid growth, no prior studies describe whether characteristics and care experiences of older dual-enrollees differ between counties with and without MLTSS. We draw on the nationally representative 2015 National Health and Aging Trends Study (NHATS) linked to the census tract of participants’ place of residence to examine differences in demographic characteristics, health, and care experiences of older dual-enrollees with LTSS need (n=462) by county-level presence of MLTSS. We broadly defined LTSS need as receiving assistance with one or more self-care or mobility activity or having probable dementia. More than half (56.9%) of older dual-enrollees with LTSS need lived in counties with MLTSS. Hispanic/other individuals were three times more likely to live in counties with (versus without) MLTSS presence (54.2% vs. 15.8%, p<0.0001) while individuals living in rural regions less likely to live in counties with MLTSS presence (8.8% vs. 31.8%, p<0.05). There were no differences in age, gender, self-reported health, dementia status, or number of chronic conditions of the sample population by MLTSS presence. Similarly, no differences were observed in experiencing adverse consequences from unmet care needs or social participation restrictions by MLTSS presence. These results are a foundational step toward building evidence and understanding who MLTSS programs are serving.

